# (*S*)-Oxiracetam is the Active Ingredient in Oxiracetam that Alleviates the Cognitive Impairment Induced by Chronic Cerebral Hypoperfusion in Rats

**DOI:** 10.1038/s41598-017-10283-4

**Published:** 2017-08-30

**Authors:** Wan li, Huihui Liu, Hanjie Jiang, Chen Wang, Yongfei Guo, Yi Sun, Xin Zhao, Xin Xiong, Xianhua Zhang, Ke Zhang, Zongxiu Nie, Xiaoping Pu

**Affiliations:** 1 National Key Research Laboratory of Natural and Biomimetic Drugs, Peking University, Beijing, 100191 P. R. China; 2 Department of Molecular and Cellular Pharmacology, School of Pharmaceutical Sciences, Peking University, Beijing, 100191 P. R. China; 3 Key Laboratory of Analytical Chemistry for Living Biosystems, Institute of Chemistry, Chinese Academy of Sciences, Beijing, 100190 P. R. China; 4 Department of Pharmacy, Peking University Third Hospital, Beijing, 100191 P. R. China

## Abstract

Chronic cerebral hypoperfusion is a pathological state that is associated with the cognitive impairments in vascular dementia. Oxiracetam is a nootropic drug that is commonly used to treat cognitive deficits of cerebrovascular origins. However, oxiracetam is currently used as a racemic mixture whose effective ingredient has not been identified to date. In this study, we first identified that (*S*)-oxiracetam, but not (*R*)-oxiracetam, was the effective ingredient that alleviated the impairments of spatial learning and memory by ameliorating neuron damage and white matter lesions, increasing the cerebral blood flow, and inhibiting astrocyte activation in chronic cerebral hypoperfused rats. Furthermore, using MALDI-MSI and LC-MS/MS, we demonstrated that (*S*)-oxiracetam regulated ATP metabolism, glutamine-glutamate and anti-oxidants in the cortex region of hypoperfused rats. Altogether, our results strongly suggest that (*S*)-oxiracetam alone could be a nootropic drug for the treatment of cognitive impairments caused by cerebral hypoperfusion.

## Introduction

Dementia is a brain disease characterized by impairment in memory, cognition, behavior and the ability to perform everyday activities. In total, 47.5 million people worldwide suffer from dementia, and there are 7.7 million new cases each year^1^. Vascular dementia (VD) is the second most common form of dementia after Alzheimer disease (AD), which accounts for at least 20% of all dementia cases^2^. Over the last decade, numerous clinical studies have supported the hypothesis that chronic cerebral hypoperfusion is associated with the cognitive decline in VD. Moreover, the decrease in the cerebral blood flow is related to the memory dysfunction that is observed in VD and post-stroke hypoperfusion^3, 4^.

Permanent bilateral common carotid artery occlusion (pBCCAO, also referred to as two vessel occlusion, 2-VO) in rats is the most commonly used chronic cerebral hypoperfusion model. During the acute phase (immediate and initial 2–3 days) of this model, the cerebral blood flow rapidly declined^5–7^, resulting in abnormal levels of small molecules, including glucose, lactate, ATP and other metabolic substrates. After the acute phase, a phase of chronic hypoperfusion follows (4 days to 3 months). During this phase, the rats undergo impairments in learning and memory, neuron damages, white matter lesions, and oxidative stress^8, 9^, which resembles the impairments that occur during human aging and dementia. The preceding acute phase, which is followed by the chronic hypoperfusion phase, contributes to the final neuropathological consequences. Currently, mass spectrometry (MS) coupled with liquid chromatography (LC-MS) or gas chromatography (GC-MS) is the most commonly used technique for studying small molecules^10, 11^. However, due to the requirement of metabolite extraction, spatial information regarding metabolites is lacking using these methods. Matrix-assisted laser desorption ionization-mass spectrometry imaging (MALDI-MSI) is a label-free technique that was introduced by Caprioli in 1997^12^. MALDI-MSI can be used to map the spatial distribution of various molecules in thin tissue sections while simultaneously providing accurate information regarding complex biological processes. To date, MALDI-MSI has been widely used for the *in situ* imaging of endogenous or exogenous molecules, such as small molecules^13, 14^, lipids^15^, peptides^16^, proteins^17^, and drugs^18^. MALDI-MSI is expected to be a powerful tool for disease diagnosis and prognosis^19^, biomarker discovery^20^, and drug development^21^. For example, using MALDI-MSI coupled with a new matrix 1,5-diaminonaphthalene (1,5-DAN) hydrochloride, 19 endogenous differential metabolites were identified in the cortex and striatum regions 24 hours after the MCAO surgery^22^. These findings provide a better understanding of the complex biological processes in MCAO and establish the foundation for investigating the changes in small molecules in the brain after drug treatment.

Oxiracetam, which is a cyclic derivative of γ-aminobutyric acid (GABA) that belongs to the racetam group (Supplementary Fig. 1a), is one of the most commonly used nootropic drugs; oxiracetam is used to treat cognitive impairments and has beneficial effects on cerebrovascular impairments and multi-infarct dementia^23–25^. The main mechanism of oxiracetam is thought to directly influence energy metabolism in the brain^26^. In clinical practice, oxiracetam is used as a racemic mixture of both (*S*)-oxiracetam (Supplementary Fig. 1b) and (*R*)-oxiracetam (Supplementary Fig. 1c). The pharmacological activity is predominantly associated with only one enantiomer in a drug. The use of the active enantiomer alone may, therefore, result in an increased efficacy, simplified pharmacokinetics, and reduced drug-drug interactions and adverse effects^27^. Compared to oxiracetam, (*S*)-oxiracetam is reported to have a higher absorption rate and a slower elimination rate; (*S*)-oxiracetam can also induce long-term synaptic potentiation in rat hippocampal slices and reverse the impairment in learning and memory induced by scopolamine^28^. These data indicate that (*S*)-oxiracetam could be the active enantiomer in oxiracetam. However, little is known regarding its nootropic effects on the cognitive impairments induced by 2-VO surgery. Moreover, the effects of (*S*)-oxiracetam on ATP and other small molecules and their spatial distributions in the brain during the acute phase of 2-VO surgery remain unclear.

This study was designed to identify the active isomer in oxiracetam that alleviates the impairments in learning and memory induced by chronic cerebral hypoperfusion in 2-VO rats. The underlying mechanism was further explored by detecting the spatial distribution changes in ATP and other small molecules in the rat brain during the acute phase of the 2-VO model using MALDI-MSI.

## Results

### (*S*)-Oxiracetam alleviated the impairments in spatial learning and memory induced by 2-VO

**Figure 1:**
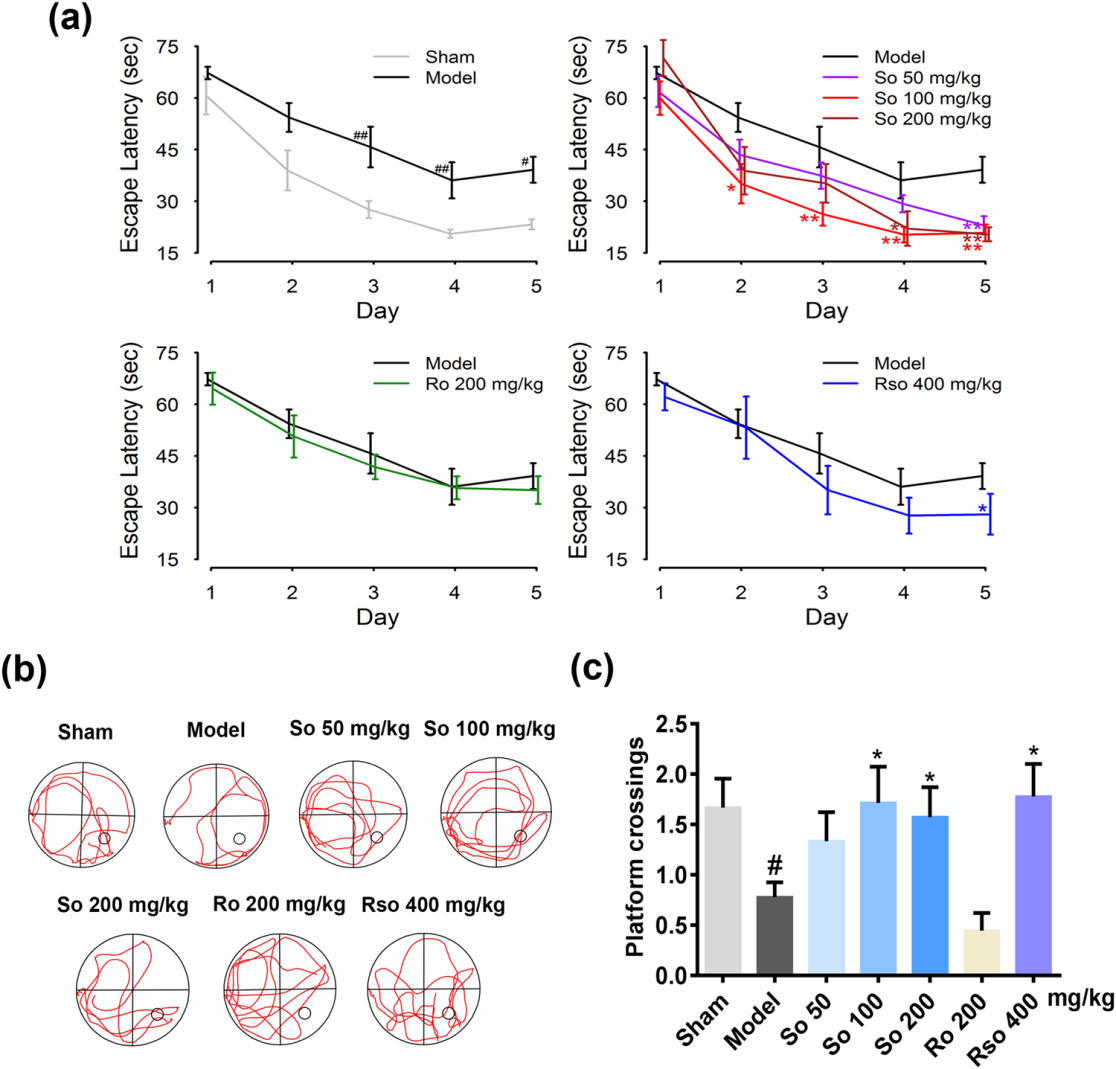
(*S*)-Oxiracetam is the active ingredient in oxiracetam that alleviates the impairments in spatial learning and memory in the Morris water maze test in the 2-VO rats. (**a**) (*S*)-Oxiracetam decreased the escape latency during the navigation training in the Morris water maze test. (**b**) Representative swim traces during the probe trail of the Morris water maze test. (**c**) (*S*)-Oxiracetam increased the platform crossings in the probe trail of the Morris water maze test. So: (*S*)-oxiracetam; Ro: (*R*)-oxiracetam; Rso: oxiracetam. The data are presented as the mean ± SEM, n = 7–9 per group. The escape latency during the navigation training was analyzed using two-way repeated measures ANOVA. The platform crossings during the probe trail were analyzed using one-way ANOVA. ^#^ *P* < 0.05, ^##^ *P < *0.01 vs. sham group; ^*^ *P* < 0.05, ^**^ *P < *0.01 vs. model group.

Six weeks after the 2-VO surgery, the Morris water maze was used to evaluate the effects of (*S*)-oxiracetam, (*R*)-oxiracetam, and oxiracetam on the impairments in spatial learning and memory (Fig. 1a,b and c). During the navigation training, the escape latency was defined as the time spent by the rats to find the hidden platform. Compared to the sham group, from the third day, the rats in the model group required a longer amount of time (*P* < 0.01, the third day; *P* < 0.01, the fourth day; *P* < 0.05 the fifth day) to find the hidden platform (Fig. 1a, upper left). Compared to the model group, the rats in the (*S*)-oxiracetam 100 mg/kg (*P* < 0.05, the second day; *P* < 0.01, the third day; *P* < 0.01, the fourth day; *P* < 0.01 the fifth day), 200 mg/kg (*P* < 0.05, the fourth day; *P* < 0.01 the fifth day) and 50 mg/kg (*P* < 0.01) groups required a shorter amount of time to find the platform from the third, fourth and fifth day, respectively (Fig. 1a, upper right). The rats in the oxiracetam 400 mg/kg (*P* < 0.05) group also required a shorter amount time to find the hidden platform on the fifth day (Fig. 1a, bottom right). However, compared to the model group, no significant changes in escape latency were observed in the (*R*)-oxiracetam group (Fig. 1a, bottom left). No significant changes in swimming speed were observed in all groups after the 2-VO surgery (Supplementary Fig. 3), indicating that the athletic ability was not changed after the 2-VO surgery.

Regarding the probe trial, representative swim traces in each group are shown in Fig. 1b. Compared to the sham group, the platform crossings were decreased in the model group (*P* < 0.05). Compared to the model group, the rats in the (*S*)-oxiracetam 100 mg/kg (*P* < 0.05) and 200 mg/kg (*P* < 0.05) and oxiracetam 400 mg/kg (*P* < 0.05) groups exhibited an increased number of platform crossings (Fig. 1c). However, compared to the model group, no significant changes in platform crossings were observed in the (*R*)-oxiracetam group (*P* > 0.05) (Fig. 1c).

### (*S*)-Oxiracetam alleviated the neuronal degeneration and white matter lesions induced by the 2-VO surgery

**Figure 2:**
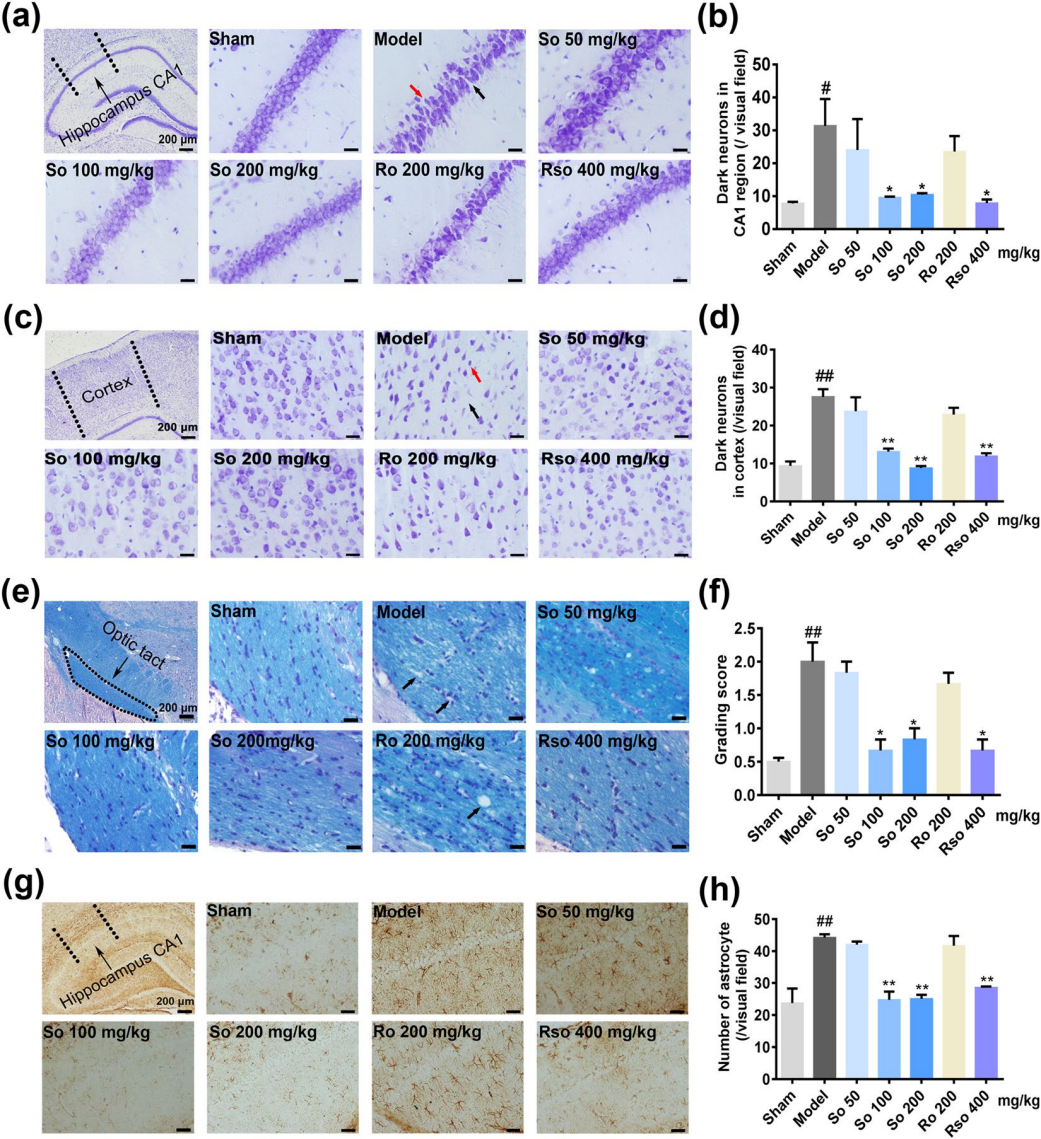
(*S*)-Oxiracetam is the active ingredient in oxiracetam that alleviates the pathological damage in the 2-VO rats. (**a**) Nissl staining in the hippocampus CA1 regions. Black arrows indicate neuronal shrinkage, and red arrows indicate neuronal loss. (**b**) (*S*)-Oxiracetam decreased the number of dark neurons in the hippocampus CA1 regions. (**c**) Nissl staining in the cortex regions. Black arrows indicate neuronal shrinkage, and red arrows indicate neuronal loss. (**d**) (*S*)-Oxiracetam decreased the number of dark neurons in the cortex regions. Dark arrow indicates vacuolation in the optic tract. (**e**) Kluver-Barrera staining in the optic tracts. (**f**) (*S*)-Oxiracetam decreased the grading scores of the white matter lesions in the optic tracts. (**g**) GFAP immunochemistry staining in the hippocampus CA1 regions. (**h**) (*S*)-Oxiracetam inhibited astrocyte activation in the hippocampus CA1 regions. So: (*S*)-oxiracetam; Ro: (*R*)-oxiracetam; Rso: oxiracetam. Scale bar = 20 μm. The data are presented as the mean ± SEM, n = 3 per group. One-way ANOVA was used to analyze the differences among the groups. ^#^ *P* < 0.05, ^##^ *P < *0.01 vs. sham group; ^*^ *P* < 0.05, **P < 0.01 vs. model group.

Seven weeks after the 2-VO surgery, Nissl staining and Kluver-Barrera staining were performed to detect the effects of (*S*)-oxiracetam on neuronal degeneration and white matter lesions. In the model group, neuronal degeneration, including neuron loss, shrinkage, and dark staining, was observed in the hippocampus CA1 and cortex regions. Representative microphotographs of the Nissl staining in the hippocampus CA1 and cortex region are shown in Fig. 2a and c, respectively. Compared to the model group, the treatment with (*S*)-oxiracetam 100 mg/kg, (*S*)-oxiracetam 200 mg/kg and oxiracetam 400 mg/kg (all *P* < 0.05, hippocampus; *P* < 0.01, cortex) decreased the number of dark neurons (Fig. 2b and d). However, the treatment with (*R*)-oxiracetam 200 mg/kg had no effects on the dark neurons (*P* > 0.05, hippocampus and cortex).

Severe rarefaction was detected specifically in the optic tract after the 2-VO surgery (Fig. 2e). Compared to the model group, the grade score of the severity of the white matter lesion in the optic tract was decreased in the (*S*)-oxiracetam 100 mg/kg (*P* < 0.05) and 200 mg/kg (*P* < 0.05) and oxiracetam 400 mg/kg (*P* < 0.05) groups but not in the (*R*)-oxiracetam 200 mg/kg (*P* > 0.05) group (Fig. 2f).

### (*S*)-Oxiracetam inhibited the activation of astrocytes in the hippocampus CA1 region induced by the 2-VO surgery

To determine the effects of (*S*)-oxiracetam on astrocyte activation, immunohistochemical analyses of GFAP were performed 7 weeks after the 2-VO surgery. Representative microphotographs of the GFAP positive cells are shown in Fig. 2g. Compared to the sham group, the number of GFAP-positive astrocytes was significantly increased in the hippocampus CA1 region in the model group (*P* < 0.01). Compared to the model group, (*S*)-oxiracetam 100 mg/kg (*P* < 0.05), (*S*)-oxiracetam 200 mg/kg (*P* < 0.05), and oxiracetam 400 mg/kg (*P* < 0.05) significantly decreased the number of GFAP-positive astrocytes in the hippocampus CA1 region (Fig. 2h). No significant changes were observed in the (*R*)-oxiracetam group (Fig. 2h).

### (*S*)-Oxiracetam increased the cerebral blood flow in rats that underwent the 2-VO surgery

**Figure 3:**
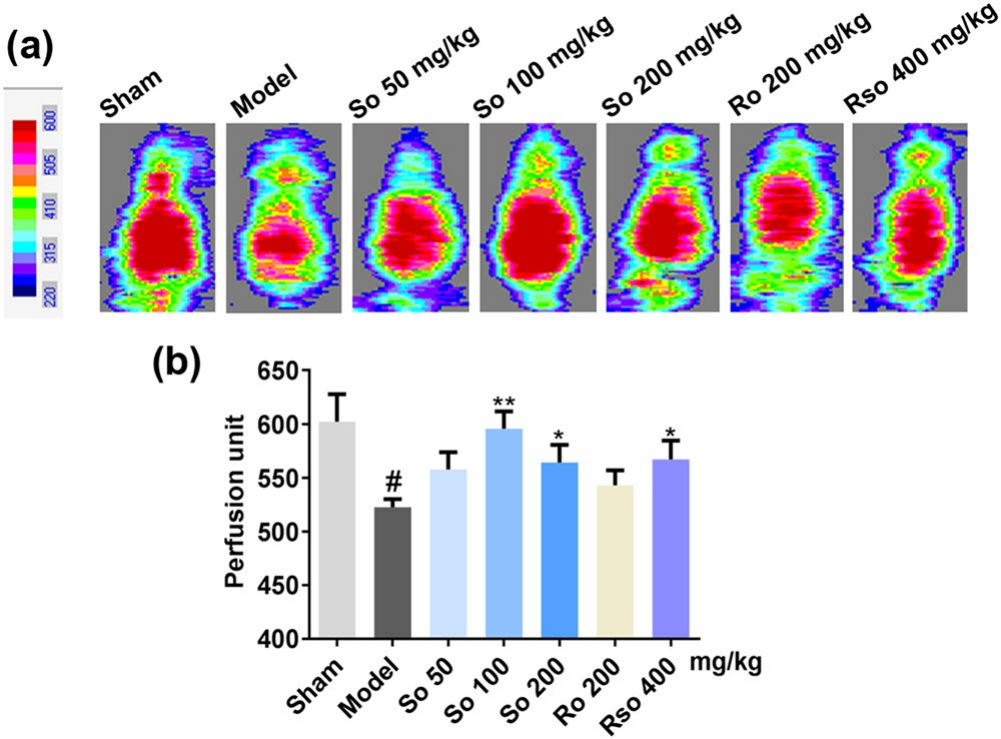
(*S*)-Oxiracetam increased the cerebral blood flow in the 2-VO rats. (**a**) Representative images of the cerebral blood flow. (**b**) (S)-Oxiracetam increased the cerebral blood flow in the cortex of the 2-VO rats. The scanner head of the laser Doppler was positioned in parallel to the skull at a distance of 15 cm to record the perfusion images. So: (*S*)-oxiracetam; Ro: (*R*)-oxiracetam; Rso: oxiracetam. The data are presented as the mean ± SEM, n = 5–6 per group. One-way ANOVA was used to analyze the differences among the groups. ^#^ *P* < 0.05, ^##^ *P* < 0.01 vs. sham group; *P < 0.05, **P < 0.01 vs. model group.

Seven weeks after the 2-VO surgery, a laser Doppler perfusion imaging system was used to determine the cerebral blood flow in the rats. Representative cerebral blood flow images are shown in Fig. 3a. The cerebral blood flow in the model group was significantly decreased compared to that in the sham group (*P* < 0.05). Compared to the model group, the cerebral blood flow was significantly increased in the (*S*)-oxiracetam 100 mg/kg (*P* < 0.01), (*S*)-oxiracetam 200 mg/kg (*P* < 0.05), and oxiracetam 400 mg/kg (*P* < 0.05) groups (Fig. 3b). However, no significant changes in cerebral blood flow were observed in the (*R*)-oxiracetam group (*P* > 0.05) (Fig. 3b).

### (*S*)-Oxiracetam exhibited nootropic effects by alleviating the abnormal metabolism of small molecules in the cortex region 2 days after the 2-VO surgery

During the acute phase of the 2-VO surgery, the concentrations of small molecules, including glucose and ATP, were decreased. In addition, the main mechanism of oxiracetam is thought to directly influence energy metabolism in the brain. In this study, to further explore the potential mechanism of (*S*)-oxiracetam during the acute phase (2 days after the surgery) of the 2-VO model, we used MALDI-MSI to investigate the changes in the spatial distribution of energy metabolism-related and other small molecules in the rat brain. In total, 15 metabolites that are involved in glucose aerobic oxidation, the TCA cycle, ATP metabolism, the glutamate-glutamine cycle, malate-aspartate shuttle, anti-oxidation, and ion homeostasis were significantly changed in the oxiracetam and (*S*)-oxiracetam groups. In total, 8 of the 13 changed metabolites were further validated by LC-MS.

#### (*S*)-Oxiracetam decreased the abnormal accumulation of glucose and citric acid and increased ATP metabolism in the cortex region 2 days after the 2-VO surgery

**Figure 4:**
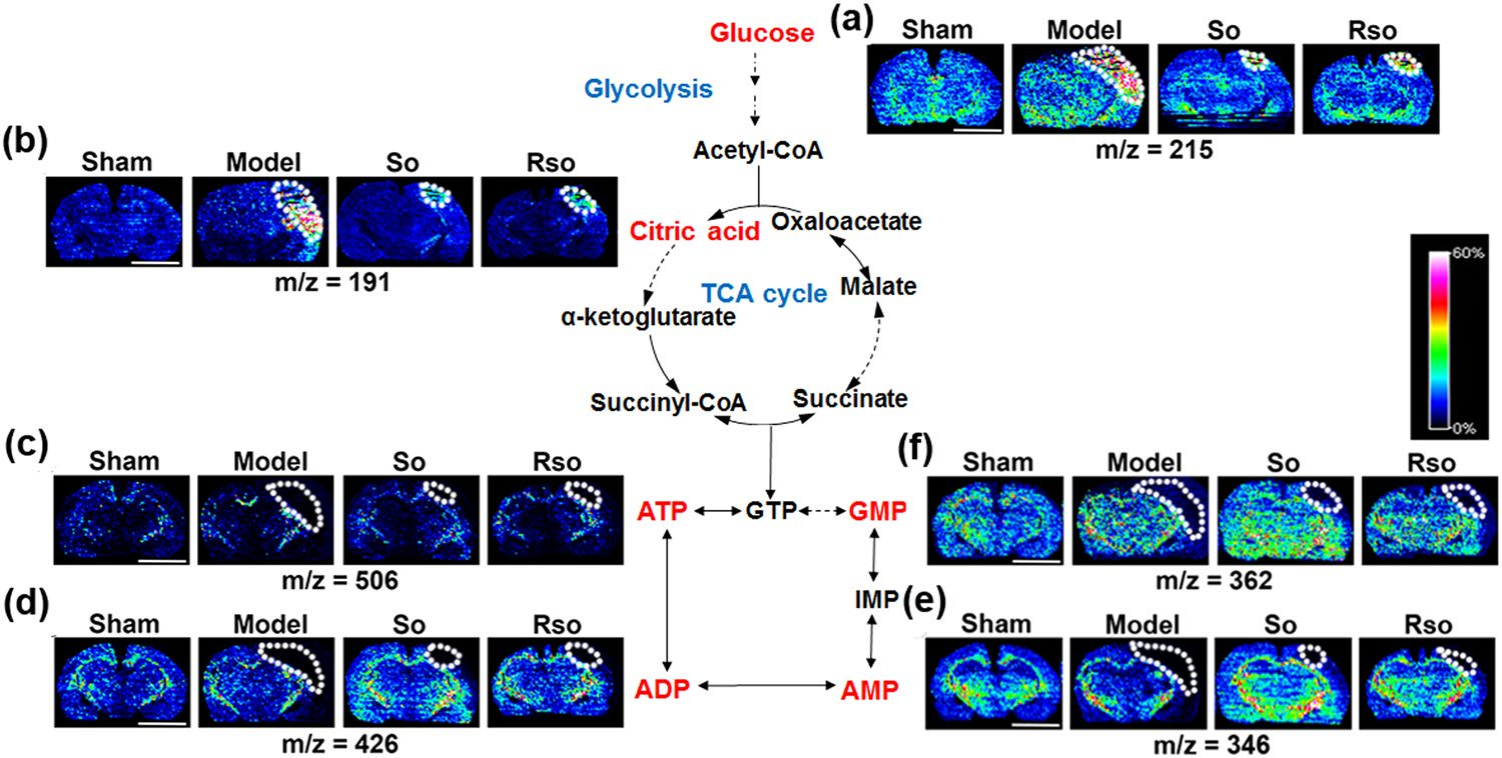
(*S*)-Oxiracetam decreased the abnormal accumulation of glucose and citric acid and increased ATP metabolism in the 2-VO rats. *In situ* MALDI MSI of (**a**) glucose; (**b**) citric acid; (**c**) ATP; (**d**) ADP; (**e**) AMP; and (**f**) GMP. The brains were rapidly removed, immediately frozen by immersion in N-hexane (−80 °C) and stored at −80 °C until use. Coronal brain sections were cut at a thickness of 10 µm. 1,5-Diaminonaphthalene (1,5-DAN) hydrochloride was used as the matrix in this experiment. Mass imaging data were acquired in negative ionization mode at a 200 μm spatial resolution. So: (*S*)-oxiracetam 200 mg/kg; Rso: oxiracetam 400 mg/kg. Scale bar = 5 mm, n = 3 per group.

Two days after the 2-VO surgery and (*S*)-oxiracetam treatment, the changes and spatial distribution of glucose, citric acid, ATP, ADP, AMP and GMP were detected in the rat brain (Fig. 4). Compared to the sham group, the content of glucose (Fig. 4a) and citric acid (Fig. 4b) in the model group was increased in the cortex region, whereas the content of ATP (Fig. 4c), ADP (Fig. 4d), AMP (Fig. 4e), and GMP (Fig. 4f) was decreased. Compared to the model group, the content of glucose and citric acid (Fig. 4a and b, respectively) in the (*S*)-oxiracetam and oxiracetam groups was decreased, whereas the content of ATP, ADP, AMP, and GMP (Fig. 4c,d,e and f, respectively) was increased in the cortex region. No significant changes in these molecules were observed in other regions of the rat brain, including the hippocampus and optic tact.

#### (*S*)-Oxiracetam increased the Glutamate-Glutamine Cycle and Malate-Aspartate Shuttle in the cortex region 2 days after the 2-VO surgery

**Figure 5:**
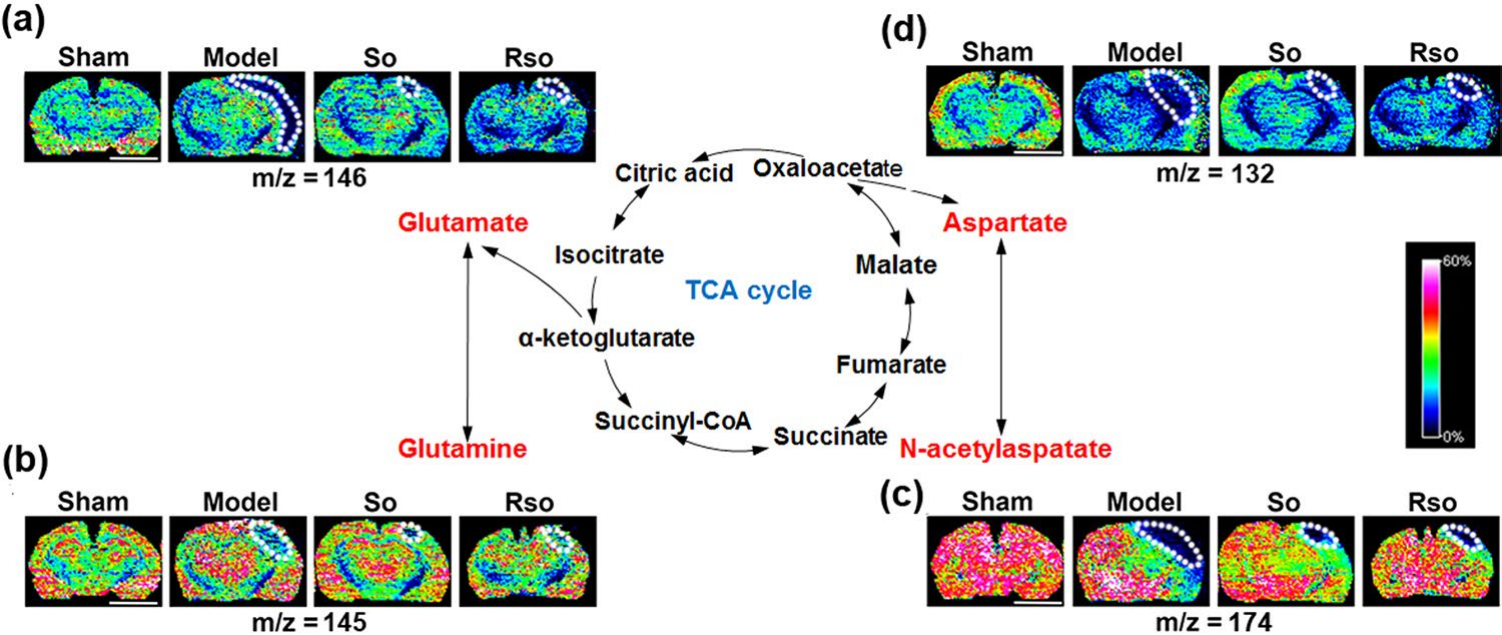
(*S*)-Oxiracetam increased the Glutamate-Glutamine Cycle and Malate-Aspartate Shuttle in the 2-VO rats. *In situ* MALDI MSI of (**a**) glutamate; (**b**) glutamine; (**c**) aspartate; and (**d**) N-acetylaspartate. The brains were rapidly removed, immediately frozen by immersion in N-hexane (−80 °C) and stored at −80 °C until use. Coronal brain sections were cut at a thickness of 10 µm. 1,5-Diaminonaphthalene (1,5-DAN) hydrochloride was used as the matrix in this experiment. Mass imaging data were acquired in negative ionization mode at a 200 μm spatial resolution. So: (S)-oxiracetam 200 mg/kg; Rso: oxiracetam 400 mg/kg. Scale bar = 5 mm, n = 3 per group.

Two days after the 2-VO surgery and (*S*)-oxiracetam treatment, the changes and spatial distribution of glutamine, glutamate, aspartate and N-acetylaspartate were detected in the rat brain (Fig. 5). Compared to the sham group, the content of glutamate (Fig. 5a), glutamine (Fig. 5b), aspartate (Fig. 5c), and N-acetylaspartate (Fig. 5d) in the cortex region of the rat brain was decreased in the model group. Compared to the model group, the content of glutamate, glutamine, aspartate, and N-acetylaspartate (Fig. 5a,b,c and d, respectively) in the cortex region was increased 2 days after the (*S*)-oxiracetam and oxiracetam treatment. No significant changes in these molecules were observed in other regions of the rat brain, including the hippocampus and optic tact.

#### (*S*)-Oxiracetam increased the content of anti-oxidants in the cortex region 2 days after the 2-VO surgery

**Figure 6:**
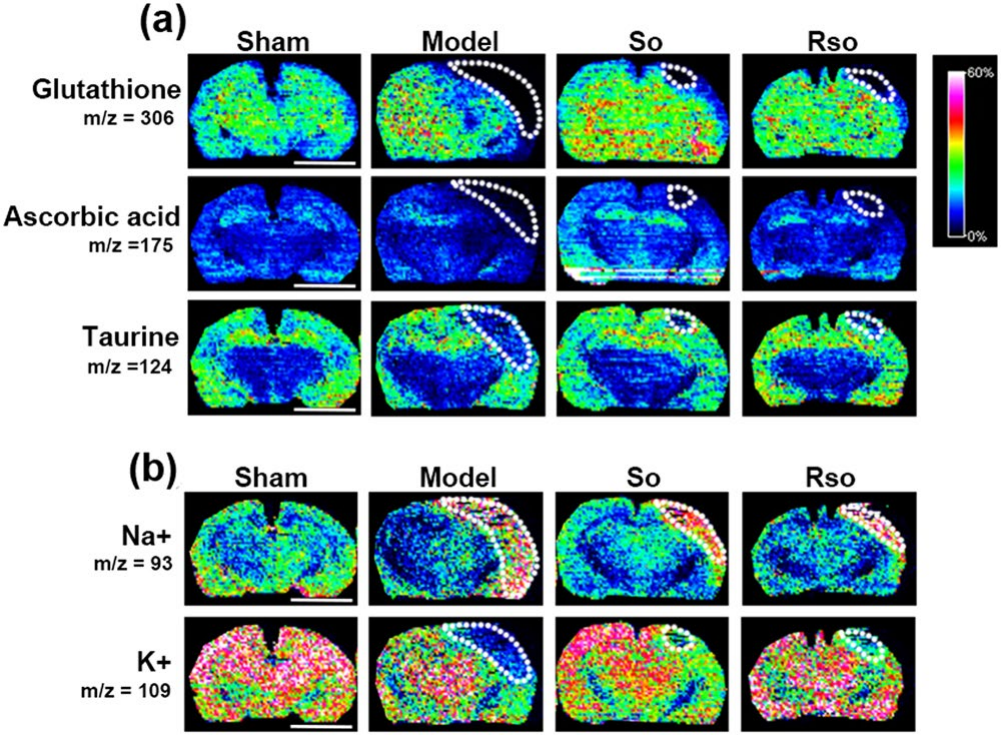
(*S*)-Oxiracetam increased the content of anti-oxidants and maintained the homeostasis of Na^+^ and K^+^ in the 2-VO rats. *In situ* MALDI MSI of (**a**) glutathione, ascorbic acid and taurine, and (**b**) Na^+^ and K^+^ . The brains were rapidly removed, immediately frozen by immersion in N-hexane (−80 °C) and stored at −80 °C until use. Coronal brain sections were cut at a thickness of 10 µm. 1,5-Diaminonaphthalene (1,5-DAN) hydrochloride was used as the matrix in this experiment. Mass imaging data were acquired in negative ionization mode at a 200 μm spatial resolution. So: (*S*)-oxiracetam 200 mg/kg; Rso: oxiracetam 400 mg/kg. Scale bar = 5 mm n = 3 per group.

**Figure 7:**
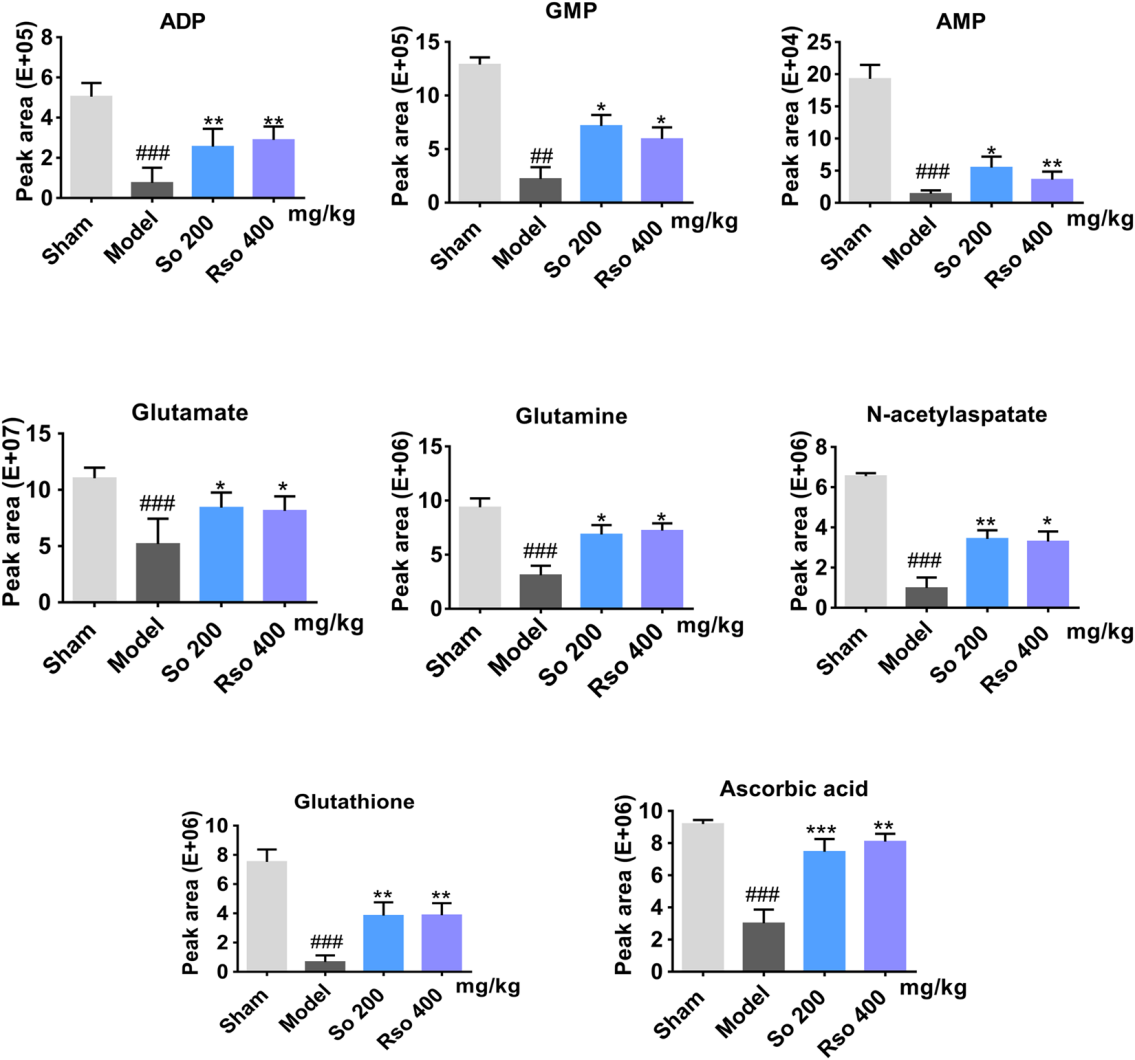
(*S*)-Oxiracetam alleviated the abnormal metabolism of small molecules in the cortex of the 2-VO rat as revealed by LC-MS/MS. A quantitative analysis of the metabolites in the rat brain was performed using LC-MS/MS in multiple reaction monitoring mode via an electrospray ionization source in the negative ion mode. Peak area counts were recorded for each metabolite included in the analysis. So: (*S*)-oxiracetam; Rso: oxiracetam. The data are presented as the mean ± SEM, n = 5–6 per group. One-way ANOVA was used to analyze the differences among the groups. ^##^ *P* < 0.01, ^###^ *P* < 0.001 vs. sham group; *P < 0.05, **P < 0.01 vs. model group.

Two days after the 2-VO surgery and (*S*)-oxiracetam treatment, the content and spatial distribution of glutathione, ascorbic acid, and taurine were detected in the rat brain (Fig. 6). Compared to the sham group, the levels of the anti-oxidants, including glutathione (Fig. 7a), ascorbic acid (Fig. 6a), and taurine (Fig. 6a), in the cortex regions were decreased in the model group. Compared to the model group, these anti-oxidants were increased in the cortex region of the oxiracetam and (*S*)-oxiracetam groups (Fig. 6a). No significant changes in these molecules were observed in (*R*)-oxiracetam groups and in the hippocampus and optic tract.

#### (*S*)-Oxiracetam maintained the homeostasis of Na^+^ and K^+^ in the cortex region 2 days after the 2-VO surgery

Two days after the 2-VO surgery and (*S*)-oxiracetam treatment, the content and spatial distribution of Na^+^ and K^+^ were detected in the rat brain. Compared to the sham group, the content of Na^+^ (Fig. 6b) was increased, and the content of K^+^ (Fig. 6b) was decreased in the cortex region in the model group. Compared to the model group, the content of Na^+^ (Fig. 6b) was decreased, and the content of K^+^ (Fig. 6b) was increased after the treatment with oxiracetam and (*S*)-oxiracetam.

#### Eight of the 15 changed metabolites were further validated by LC-MS/MS

LC-MS/MS was performed to validate the 15 changed metabolites that were identified by MALDI-MSI. As shown in Fig. 7 and Supplementary Table 2, compared to the sham group, except for glucose, 12 metabolites (*P* < 0.05, citric acid and taurine; *P* < 0.01, ATP and ADP; *P* < 0.001, AMP, GMP, glutamate, glutamine, aspartate, N-acetylaspartate, glutathione and ascorbic acid) were significantly changed in the model group, which was consistent with the MALDI MSI results. Furthermore, compared to the model group, 8 of the 13 metabolites were also significantly changed in the (*S*)-oxiracetam and oxiracetam groups as follows: ADP and glutathione (*P* < 0.01 for (*S*)-oxiracetam and oxiracetam); AMP, GMP, glutamine and glutamate (*P* < 0.05 for (*S*)-oxiracetam and oxiracetam); N-acetylaspartate (*P* < 0.01, (*S*)-oxiracetam; *P* < 0.05, oxiracetam); and ascorbic acid (*P* < 0.001, (*S*)-oxiracetam; *P* < 0.01, oxiracetam). These metabolites were involved in ATP metabolism, the Glutamine-Glutamate cycle and anti-oxidants.

## Discussion

In this study, we used the 2-VO model to investigate the stereoselective pharmacological activities of oxiracetam. We demonstrated for the first time that (*S*)-oxiracetam, but not (*R*)-oxiracetam, was the active ingredient in oxiracetam that alleviated the impairments in learning and memory, ameliorated the pathological damages, and increased the cerebral blood flow in the 2-VO rats. Its nootropic effects could be related to the inhibition of astrocyte activation in the hippocampus CA1 region during the chronic phase. During the acute phase in the 2-VO rats, (*S*)-oxiracetam acts by rescuing the abnormal metabolism of ATP and increasing the content of glutamate, glutamine and other small molecules in the cortex region.

Previous animal experiments have demonstrated that chronic cerebral hypoperfusion could induce impairments in learning and memory in 2-VO rats^29^. The Morris water maze is most frequently used to measure the spatial learning capacity in rats undergoing chronic cerebral hypoperfusion. In this study, using the Morris water maze test, we found that only (*S*)-oxiracetam and oxiracetam ameliorated the impairments in learning and memory (decreased escape latency and increased platform crossing) induced by the 2-VO surgery. This finding was consistent with a previous study in which (*S*)-oxiracetam was shown to be the active ingredient in oxiracetam that reversed the cognitive impairments induced by scopolamine. In this study, a dose-dependent effect of (*S*)-oxiracetam was not obvious, which may be due to the large individual variability in the behavioral test. In addition, the absorption of (*S*)-oxiracetam may be saturated at high doses^30^, which might have influenced the nootropic effects of (*S*)-oxiracetam.

Chronic cerebral hypoperfusion can result in severe neuronal damage in the hippocampus, cerebral cortex, and white matter (WM). Seven weeks after the 2-VO surgery, neuronal damage, including neuronal loss, shrinkage, and dark staining, was observed in the hippocampus and cortex. White matter lesions, including vacuolation and a disarrangement of the myelin fibers in the optical tract, were also detected. Our results showed that (*S*)-oxiracetam, but not (*R*)-oxiracetam, ameliorated the neuronal damage and white matter lesions. These histological changes were consistent with the cognitive impairments revealed by the Morris water maze test.

The cerebral blood flow plays an important role in maintaining normal brain functioning. A decreased cerebral blood flow was observed in dementia patients. Previous studies have shown that piracetam can increase the cerebral blood flow both in ischemic cats^31, 32^ and post-stroke aphasic patients^33^. In addition, piracetam can reduce the erythrocyte adhesion to the vascular endothelium, hinder vasospasm, and facilitate microcirculation^34^. Our results showed that the S-isomer increased the cerebral blood flow after the 2-VO surgery. However, (*R*)-oxiracetam had no such beneficial effects, indicating that (*S*)-oxiracetam, but not (*R*)-oxiracetam, was the active ingredient in oxiracetam that increased the cerebral blood flow in the chronic cerebral hypoperfused rats. Chronic reduction in cerebral blood flow in the adult rat is associated with a late-emerging CA1 cell loss and memory dysfunction. CA1 neurons begin to degenerate after several weeks of reduced energy availability due to the 2-VO, and this degeneration impairs memory. Since reduced neuronal energy metabolism is associated with the progressive neurodegeneration in disorders, such as Alzheimer’s disease^29^, the effect of oxiracetam on cerebrovascular impairment was investigated in rats. Oxiracetam administered after the re-perfusion at a dose of 100 mg/kg (i.v.) accelerated recovery. Oxiracetam is thought to have a protective effect against cerebrovascular impairment^24^. The improved cerebral blood flow after the (S)-oxiracetam treatment may be caused by the increased neuronal energy metabolism such as ATP metabolism and its protective effect in the cerebrovascular impairment. However, the specific mechanisms require further investigation.

Astrocyte activation is one of the major producers of inflammatory mediators in the nervous system^35^. Previous studies have shown that astrocytes are activated during the chronic phase^36^ of the 2-VO model. Activated astrocytes can cause neuronal damage and a blood brain barrier dysfunction by releasing neurotoxic molecules, including pro-inflammatory factors, reactive oxygen species, and reactive nitrogen species^37, 38^. In the 2-VO model, the astrocyte activation is closely associated with the cognitive impairments^39, 40^, and the control of astrocyte activation is likely an important therapeutic target^41, 42^. In addition, astrocytes play an important role in the dynamic regulation of the cerebral circulation^43^. In this study, we found that many reactive astrocytes that were labeled with GFAP were activated in the hippocampus CA1 region. In addition, (S)-oxiracetam alone inhibited the astrocyte activation. These results suggested that the nootropic effects of (S)-oxiracetam in the 2-VO rats might be related to the inhibition of astrocyte activation.

To further explore the underlying mechanisms of (*S*)-oxiracetam’s nootropic effects during the acute phase, we investigated the spatial distribution changes in small molecules in the brain tissues using MALDI-MSI. MALDI-MSI has been extensively used to analyze small molecules in biological samples by providing direct 2D visualization of the metabolic profiles. In this study, we systematically investigated the changes in the spatial distribution of ATP and other small molecules during the acute phase of the 2-VO rat brain. Our results showed that (*S*)-oxiracetam decreased the abnormal accumulation of glucose and citric acid and increased ATP metabolism. In addition, we demonstrated for the first time that (*S*)-oxiracetam increased the Glutamate-Glutamine Cycle, Malate-Aspartate Shuttle and the content of anti-oxidants and maintained the homeostasis of Na^+^ and K^+^ in the cortex region of the 2-VO rats.

Oxiracetam can cross the blood-brain barrier and distribute in the septum, hippocampus, cerebral cortex, and striatum^44^. (S)-oxiracetam has distribution profiles similar to racemic oxiracetam^45^. In addition, it has both higher absorption and slower elimination rates than racemic oxiracetam^30^, suggesting that (S)-oxiracetam might have a more favorable pharmacokinetic profile that can decrease toxicology risks and clinical dosage when developed as a new drug^30, 45^. The cortex region is more vulnerable to cerebral ischemia than other regions of the brain^46^. In addition, the regions of ischemic stroke mainly appear in the right hemisphere of the human brain^47^, which could be attributed to the more frequent use of the right hand, which leads to an increased blood supply in the left hemisphere^48, 49^, thereby leaving the right hemisphere more vulnerable to ischemic stroke. Furthermore, it could be caused by the asymmetrical insula and parietal control of the autonomic nervous system and alterations in the norepinephrine levels^47^. In this study, we also observed that the ischemic regions were mainly located in the cortex region in the right rat brain, indicating that laterality might also exist in rats^50, 51^. These results were also consistent with a previous 2-OV study reported by Sónia Sá Santos *et al*., where only one side of the brain is affected in the representative figure^52^. And (*S*)-oxiracetam ameliorated the abnormal metabolism of small molecules in the right cortex region.

Glucose is the key source of energy in the brain, and citric acid is the first mediator of the Tricarboxylic acid (TCA) cycle. Previous studies have shown that oxiracetam increases glucose utilization in cerebral ischemic rats^53^. In this study, the MSI results showed that the levels of glucose and citric acid increased after the 2-VO surgery. Consistently with a previous study, we found that (*S*)-oxiracetam decreased the abnormal accumulation of glucose and citric acid in the cortex region.

Glucose aerobic oxidation coupled with phosphorylation is the major pathway of ATP generation. ATP can be catabolized to ADP, AMP, and eventually uric acid following two main catabolic routes^54^. The restricted oxygen and glucose delivery caused by the 2-VO surgery interrupted mitochondrial oxidative phosphorylation and ATP synthesis. In this study, ATP, ADP and AMP were decreased in the model group. A previous study has shown that oxiracetam increases the ATP content in cultured astrocytes^26^ and improves the ratio of brain adenosine triphosphate/adenosine diphosphate (ATP/ADP) in the brain, and our study found that (*S*)-oxiracetam and oxiracetam increased the content of ATP and its downstream products ADP and AMP. These results suggested that (*S*)-oxiracetam and oxiracetam could rescue the interrupted mitochondrial oxidative phosphorylation and subsequent ATP metabolism.

In this study, the MSI results showed that the levels of glucose and citric acid increased, whereas the levels of ATP, ADP, and AMP decreased after the 2-VO surgery. The increase in glucose caused by the 2-VO surgery was likely due to the lower glucose utilization in the brain as suggested by a previous report^53^. The increased levels of citric acid and decreased levels of downstream products, including glutamate and aspartate, could have been caused by the arrest of the TCA cycle at the step from citrate to α-ketoglutarate. Furthermore, previous studies have shown that mitochondrial enzymes, such as aconitase and 2-oxoglutarate dehydrogenase, were inactivated^55, 56^, and the activity of citrate synthase was unchanged after ischemia^57^.

Glutamate-mediated excitotoxicity plays an important role in the pathogenesis of cerebral ischemia^58^. L-Aspartate is another excitatory neurotransmitter that plays a key role in the malate-aspartate shuttle that transfers NADH in the cytosol through the inner membrane to the mitochondria for oxidative phosphorylation and ATP synthesis. N-acetylaspartate (NAA), which is an indicator of neuronal damage and mitochondrial dysfunction, is synthesized in the mitochondria from aspartate and acetyl-CoA by the enzyme L-aspartate N-acetyltransferase^57^. A previous study has shown that piracetam decreases the release of glutamate and aspartate in cortical cells upon oxygen and glucose deprivation. However, it was unknown whether oxiracetam had effects on glutamate and aspartate. In this study, the levels of glutamine, glutamate, aspartate, and N-acetylaspartate (NAA) decreased after the 2-VO surgery. The treatment with (*S*)-oxiracetam and oxiracetam increased the content of these metabolites, indicating that oxiracetam and (*S*)-oxiracetam also have effects on glutamate and aspartate.

During the ischemic period, the restricted delivery of oxygen impairs ATP synthesis and causes the continuous production of reactive oxygen species^59^, which can result in necrosis, apoptosis, and eventually neuronal death. Glutathione (GSH) and ascorbic acid (AA) are important anti-oxidants that can directly scavenge ROS in the brain. Although taurine is not a characteristic scavenger of ROS, it exhibits anti-oxidant activity through several indirect mechanisms, including reducing ROS production, limiting ROS activation, and interfering with ROS-producing inflammatory reactions^60^. Previous studies have reported that piracetam reverses the cognitive dysfunction and reduces the oxidative stress induced by propoxur and phosphamidon in the brain^61, 62^. However, it was unknown whether oxiracetam had anti-oxidative stress effects. In this study, the content of glutathione, ascorbic acid, and taurine decreased after the 2-VO surgery. The treatment with (*S*)-oxiracetam and oxiracetam increased the content of glutathione, ascorbic acid, and taurine in the cortex region, which is consistent with their anti-oxidant effects in which the SOD activity increased, and the MDA content decreased during the chronic phase (Supplementary Fig. 4).

Na^+^ and K^+^ are important inorganic ions in the brain. These ions are responsible for maintaining the membrane potential and are essential for neuronal activities. Na^+^- K^+^-ATPase and Na^+^-K^+^-2Cl^−^cotransporter (NKCC) are two important transporters/channels that maintain the intracellular and extracellular Na^+^ and K^+^ concentrations. After the occlusion of the cerebral artery, the supply of oxygen and glucose is not sufficient to power the Na/K ATPase. The increased concentration of Na^+^ and decreased concentration of K^+^ in the brain tissue result in necrosis^63, 64^. The α-amino-3-hydroxy-5-methyl-4-isoxazolepropionic acid (AMPA) receptor is a glutamate ionotropic transmembrane receptor that mediates rapid synaptic transmission in the brain^65^. The principal ions fated by the AMPA receptor are Na^+^ and K^+^. Oxiracetam is reported to act as a positive allosteric modulator of the AMPA receptor^66^. However, whether oxiracetam has effects on Na^+^ and K^+^ is unknown. Our results showed that the concentration of Na^+^ increased, and the concentration of K^+^ decreased in the cortex region after the 2-VO surgery. The treatment with oxiracetam and (*S*)-oxiracetam rescued the abnormal ion content, which might have been caused by the increased activity of abluminal Na/K-ATPase, the decreased activity of luminal endothelial NKCC^67^, and the modulation of the AMPA receptor. However, the specific mechanisms require further investigation.

This is the first report describing the precise distribution of small molecules after (*S*)-oxiracetam treatment by MALDI-MSI during the acute phase of 2-VO. The MALDI-MSI technique can be successfully applied to the pharmacological evaluation and study of mechanisms. Although previous studies have investigate different aspects of the mechanisms of (*S*)-oxiracetam, these studies lack spatial distributing information of the analyzed substances. Using MALDI-MSI, we simultaneously observed 15 changed small molecules in the right cortex region of the rat brain. This finding allowed us obtain spatial distributing information regarding the changed small molecules after the drug treatment and a better understanding of the mechanisms of (*S*)-oxiracetam.

Of the 15 metabolites identified by MALDI-MSI, the following eight metabolites were also validated by LC-MS/MS: glutamine, glutamate, N-acetylaspartate, ascorbic acid, glutathione, ADP, AMP, and GMP. However, the existence of glucose isomers and the degradation of ATP could result in the failure of their validation using LC-MS/MS. The eight metabolites identified by the two distinct methods were related to the ATP metabolism pathway, the glutamate-glutamine cycle and anti-oxidants, which is indicative of their contribution to the nootropic effects of (*S*)-oxiracetam. ATP is the most direct source of energy in the human body. After an ischemic stroke, the decreased blood flow causes an ATP reduction and energy depletion and initiates excitotoxic mechanisms that are harmful to neurons. Glutamate is the major excitatory amino acid in the brain and plays an important role in the pathogenesis of cerebral ischemia. Ascorbic acid and glutathione are important anti-oxidants that can directly scavenge ROS in the brain. (*S*)-Oxiracetam increased ATP metabolism, the glutamate-glutamine cycle, the malate-aspartate shuttle, and the content of anti-oxidants. This finding indicates that (*S*)-oxiracetam could exert its nootropic effects by increasing the energy supply, inhibiting the toxicity of excitatory amino acids, increasing the content of anti-oxidants, and eventually reducing neuronal death.

Neuronal cell death occurs throughout the chronic cerebral hypoperfusion; during the acute phase of the 2-VO surgery, the blood flow disruptions rapidly limit the delivery of oxygen and glucose to neurons, thereby causing ATP reduction and energy depletion and initiating excitotoxic mechanisms that are deleterious to neurons^65, 68^. In turn, this process aggravates the reduction in glucose and ATP. Therefore, the decreased concentration of small molecules, such as ATP and glucose, observed after the 2VO-surgery could be due to the decreased blood flow and/or the increased neuronal death. Similarly, the increased concentration of small molecules after the (S)-oxiracetam treatment could be due to the increased cerebral blood flow and/or neuron survival. Oxiracetam acts as a positive modulator of AMPA-sensitive glutamate receptors in neurons^66^. This action increases the density of receptor binding sites for AMPA and calcium uptake^66, 69^. The AMPA receptor, which is another type of ionotropic channel, is known to mediate the rapid and immediate postsynaptic response to glutamate release and, thus, may contribute to synaptic plasticity^70^. So, the possibility that oxiracetam increases the glutamate concentration in the cortex region of 2-VO rats may be related to its positive regulation on the AMPA receptor. However, no studies have investigated whether (S)-oxiracetam increases the concentrations of small molecules by increasing neuronal survival and positive regulate AMPA receptor in the cortex region using the 2-VO model. Its detailed mechanism needs to be further explored in the future.

In conclusion, our study demonstrated that (*S*)-oxiracetam, but not (*R*)-oxiracetam, was the active ingredient in oxiracetam that alleviated the cognitive impairments in chronic cerebral hypoperfused rats. (*S*)-Oxiracetam exerted its nootropic effects by decreasing astrocyte activation and rescuing the abnormal metabolism of small molecules in the brain tissues during the acute phase in 2-VO rats. Therefore, (*S*)-oxiracetam alone might be a nootropic drug suitable for the treatment of dementia caused by chronic cerebral hypoperfusion.

## Material and Methods

### Animals

Male Wistar rats weighing 230 to 250 g were purchased from Beijing Vital River Laboratory Animal Technological Company, confirmation number SCXK (JING) 2012-0001 from the local animal committee. All animals were housed in individually ventilated cages (temperature 25 ± 1 °C, relative humidity 50% ± 10%, 12-hour light/dark cycle) with free access to drinking water and a pelleted basal diet (Beijing Keaoxieli Fodder Co. Ltd.). All animal experiments were conducted according to the principles of the NIH Guide for the Care and Use of Laboratory Animals and were approved by the Ethics Committee for Laboratory Animal Care and Use of Peking University Health Science Center. All efforts were made to minimize the suffering of the animals used in this study.

### Surgical procedures

The animals were anesthetized with pentobarbital sodium (60 mg/kg, i.p.), and a cervical incision was made to expose and separate the common carotid artery from the carotid sheath and vagus nerve. For the chronic phase study, the right common carotids were ligated first, and the left common carotids were ligated 1 week later. Both the right and left common carotids were double-ligated using 5–0 silk sutures and cut between ligations. For the acute phase study, the bilateral common carotid arteries were double-ligated simultaneously using 5–0 silk sutures and cut between ligations. The rats in the sham group were subjected to the same procedure without the arterial ligation.

### Drug preparation and administration

Oxiracetam (Huan Jianlang Pharmaceutical Co., Ltd.), (*S*)-oxiracetam and (*R*)-oxiracetam (Harbin Median Pharmaceutical Co., Ltd.) were diluted with a 0.9% sodium chloride solution and filtered through a 0.22-µm membrane. After the surgery, the drugs were intravenously administered for 6 weeks in the chronic phase study and 2 days in the acute phase study, respectively. Oxiracetam is used as a racemic mixture of both (S)-oxiracetam (Supplementary Fig. 1b) and (R)-oxiracetam (Supplementary Fig. 1c). To investigate whether (S)-oxiracetam is the active ingredient in Oxiracetam, we used 200 mg/kg (S)-oxiracetam and 200 mg/kg (R)-oxiracetam to determine whether they have the same effects as 400 mg/kg oxiracetam. Diagrammatic representations of the experimental protocols of the chronic phase and acute phase studies are shown in Supplementary Fig.  2a and b, respectively.

### Experiment 1: Evaluation of the nootropic effects of S-oxiracetam during the chronic phase of the 2-VO model

#### Grouping

In total, 140 male Wistar rats were quarantined for 7 days and then randomized according to body weight into a control group and six experimental groups as follows: (1) Sham group (Sham); (2) Bilateral common carotid artery occlusion group (Model); (3) (S)-oxiracetam 50 mg/kg group (low dose group, So-L); (4) (S)-oxiracetam 100 mg/kg group (medium dose group, So-M); (5) (S)-oxiracetam 200 mg/kg group (high dose group, So-H); (6) (*R*)-oxiracetam 200 mg/kg group (Ro); and (7) Oxiracetam 400 mg/kg group (Rso). Groups 2–7 underwent the 2-VO surgery.

#### Morris water maze test

Six weeks after the 2-VO surgery, the Morris water maze test was performed to evaluate spatial learning and memory. The experimental conditions of the Morris water maze test are shown in Supplementary material. The data were acquired using a video camera connected to a computerized tracking system (SLY-ETS, version 1.3) fixed above the center of the water maze.

During the navigation test, each rat underwent four trials on each testing day for 5 consecutive days with 15 min intervals between each trial. During each trial, the rat was allowed 90 s to find the hidden platform. If the animal failed to find the platform within 90 s, it was guided to the platform by the experimenter, and the escape latency time was recorded as 90 s. The rat was allowed to remain on the platform for 30 s before being removed.

A spatial probe trial was used to measure the retention of spatial reference memory after the navigation test. The hidden platform was removed, and the rat was allowed to search for the absent platform for 60 s before being removed from the pool. The time spent in the target quadrant and the number of platform crossings were recorded, and the recall of the platform position in each group was compared.

#### Histological analysis

After the cerebral blood flow measurement, three rats from each group were randomly selected for the histological analysis. After being anesthetized with pentobarbital-sodium (60 mg/kg i.p.), the rats were perfused with 0.9% sodium chloride and then 4% paraformaldehyde (PFA). The brains were quickly removed, fixed in 4% PFA for at least one day, and embedded in paraffin. Coronal brain sections were cut at a 10 µm thickness for the Nissl and Kluver-Barrera staining. Using the Nissl staining, the neurons that exhibited shrinkage and nucleus diffusion in each delineated region were counted according to the physical dissector counting rule^71, 72^. Using the Kluver-Barrera staining, the severity of the white matter lesion was graded as follows: grade 0 (normal), grade 1 (disarrangement of the nerve fibers), grade 2 (formation of marked vacuoles), and grade 3 (disappearance of myelinated fibers)^73^.

#### Immunohistochemical analysis of glial fibrillary acidic protein (GFAP)

Coronal brain sections (10 μm thickness) used in the immunochemistry assays were placed on polylysine-coated slides (ZLI-9501, Zhongshan Goldenbridge Biotechnology, China). The de-paraffinized sections were immersed in boiled 10 mM sodium citrate buffer (pH 6.0) and maintained at a sub-boiling temperature for 30 min. Then, the peroxidase activity was inactivated by incubation with a 3% H_2_O_2_ solution for 10 min at room temperature. Then, the sections were incubated with a primary anti-GFAP antibody (3670 S, 1:50, Cell Signaling Technology) in a moist chamber at 4 °C overnight. Negative controls were established by exchanging the primary antibody with PBS. The sections were then reacted using a Polink-2 Plus IHC Detection System (PV9005, Zhongshan Goldenbridge Biotechnology, China) according to the manufacturers’ instructions. Finally, the samples were incubated in diaminobenzidine (DAB) until a brown reaction product was observed (approximately 30 s).

Photomicrographs were taken with equal exposure under an Olympus microscope (Olympus IX71; 4 × , 10 × , 20 × or 40 × magnification) coupled to a computer running Olympus Images Olympus Entry software for Windows. Three visual fields were randomly selected for the quantitative analysis, which was performed independently by two observers.

#### Cerebral blood flow measurement

The cerebral blood flow (CBF) was detected using a laser Doppler perfusion imaging system (PeriScan PIM3 System; PERIMED, Stockholm, Sweden) 7 weeks after the 2-VO surgery according to the manufacturers’ instructions. The rats were anesthetized with pentobarbital sodium (60 mg/kg, i.p.). An incision was made through the scalp to expose the skull, and the periosteal connective tissue was removed using a sterile cotton swab. The scanner head of the laser Doppler was positioned in parallel to the skull at a distance of 15 cm to record the perfusion images. For each rat, the perfusion image was collected three times.

### Experiment 2: Mass spectrometry imaging of small molecules during the acute phase of the 2-VO model

#### Grouping

In total, 40 male Wistar rats were quarantined for 7 days and then randomized according to their body weight into a control group and three experimental groups as follows: (1) Sham group (Sham); (2) Bilateral common carotid artery occlusion group (Model); (3) (*S*)-oxiracetam 200 mg/kg group (So-H); and (4) Oxiracetam 400 mg/kg group (Rso). Groups 2–4 underwent the 2-VO surgery.

#### Tissue preparation

After the rats were anesthetized with an overdose of sodium pentobarbital (60 mg/kg, i.p.), the rats were perfused with 0.9% sodium chloride, and the brains were rapidly removed, immediately frozen by immersion in N-hexane (−80 °C) and stored at −80 °C. The frozen brain tissues were cut using a cryostat-microtome (Leica CM1950, Leica Microsystems). Coronal brain sections were cut at a thickness of 10 µm. The tissue sections were transferred by thaw mounting onto conductive indium tin oxide (ITO) glass slides (Bruker Daltonics). The glass slides were then stored under a vacuum in a desiccator for approximately 1 hour before the matrix application. The matrix solution, i.e., 1,5-diaminonaphthalene (1,5-DAN) hydrochloride in 50% ethanol/water, was prepared as previously described^22^ and sprayed on the tissue sections that were mounted on ITO coated glass slides using an automatic matrix sprayer (ImagePrep, Bruker Daltonics) while ensuring an homogeneous matrix coverage over the entire tissue surface.

#### Mass spectrometry imaging of small molecules in the brain tissue sections

An Ultraflextreme MALDI-TOF/TOF MS (Bruker Daltonics, Billerica, MA) equipped with a SmartBeam Nd: YAG 355 nm laser was utilized for the MALDI analysis. The data were acquired in negative ionization mode with a 200 μm spatial resolution (200 laser shots), and the signals between *m*/*z* 80 and 1,000 were collected. The laser spot size of the instrument used in the imaging experiment was approximately 50 µm; the laser power was optimized at the start of each run and then fixed for the whole experiment. The regions of interest (ROI) were manually created by drawing an outline of each brain slice both in the optical image and the MSI data image. All MSI data were normalized according to the total ion current, and the signal intensity of each image in the figure is represented as the normalized intensity. The methods used to identify and further confirm the metabolites were performed according to a previous study^22^.

#### Quantitative analysis of small molecules in brain tissue homogenates using liquid chromatography tandem mass spectrometry (LC-MS/MS)

For the LC-MS, the rat brain samples were prepared as previously described^22^. In brief, the quantitative analysis of the metabolites in the rat brain was performed using an AB Sciex QTrap 5500 mass spectrometer in multiple reaction monitoring (MRM) mode via an electrospray ionization (ESI) source in negative ion mode. A 2-μL aliquot of each sample was injected directly through a bypass into MS without chromatography separation. The optimized instrument conditions were as follows: the electrospray voltage was maintained at −4500 V; the turbo ion spray source temperature was set at 500 °C; nitrogen was used as the collision gas; curtain gas (CUR), and nebulizer gas (GS1), and turbo-gas (GS2) were set at 30, 50, and 50 psi, respectively; the optimized parameters, such as the quantification ion transitions and collision energy (CE) for each compound, are summarized in Supplementary Table 1. The peak area was recorded for each metabolite included in the analysis.

### Statistical analysis

The numerical data are presented as the mean ± SEM. The differences in the escape latency in the Morris water maze test among the groups were analyzed using two-way analysis of variance (ANOVA) with repeated measures. One-way ANOVA was used to analyze the group differences in the probe trial, cerebral blood flow, biochemical analysis, number of dark neurons in the cortex and hippocampus, grading score, number of astrocyte and small molecule changes. Statistical significance was set at α = 0.05 (two sided).

## Electronic supplementary material

Supplementary data

## References

[1] WHO. Dementia http://www.who.int/mediacentre/factsheets/fs362/en/, (Data of access: 05/2017) (2017).

[2] Iadecola C (2013). The pathobiology of vascular dementia. Neuron.

[3] Komatani A (1988). Assessment of demented patients by dynamic SPECT of inhaled xenon-133. Journal of nuclear medicine: official publication, Society of Nuclear Medicine.

[4] Ohta H, Nishikawa H, Kimura H, Anayama H, Miyamoto M (1997). Chronic cerebral hypoperfusion by permanent internal carotid ligation produces learning impairment without brain damage in rats. Neuroscience.

[5] Otori T (2003). Long-term measurement of cerebral blood flow and metabolism in a rat chronic hypoperfusion model. Clinical and experimental pharmacology & physiology.

[6] Tsuchiya M, Sako K, Yura S, Yonemasu Y (1993). Local cerebral glucose utilisation following acute and chronic bilateral carotid artery ligation in Wistar rats: relation to changes in local cerebral blood flow. Experimental brain research.

[7] Plaschke K (2005). Aspects of ageing in chronic cerebral oligaemia. Mechanisms of degeneration and compensation in rat models. Journal of neural transmission.

[8] Farkas E (2004). Diazoxide and dimethyl sulphoxide prevent cerebral hypoperfusion-related learning dysfunction and brain damage after carotid artery occlusion. Brain research.

[9] Farkas E, Luiten PG, Bari F (2007). Permanent, bilateral common carotid artery occlusion in the rat: a model for chronic cerebral hypoperfusion-related neurodegenerative diseases. Brain research reviews.

[10] Werner E (2008). Mass spectrometry-based metabolomics: accelerating the characterization of discriminating signals by combining statistical correlations and ultrahigh resolution. Analytical chemistry.

[11] Major HJ, Williams R, Wilson AJ, Wilson ID (2006). A metabonomic analysis of plasma from Zucker rat strains using gas chromatography/mass spectrometry and pattern recognition. Rapid communications in mass spectrometry: RCM.

[12] Caprioli RM, Farmer TB, Gile J (1997). Molecular imaging of biological samples: localization of peptides and proteins using MALDI-TOF MS. Analytical chemistry.

[13] Nilsson A (2010). Fine mapping the spatial distribution and concentration of unlabeled drugs within tissue micro-compartments using imaging mass spectrometry. PloS one.

[14] Hsieh Y (2006). Matrix-assisted laser desorption/ionization imaging mass spectrometry for direct measurement of clozapine in rat brain tissue. Rapid communications in mass spectrometry: RCM.

[15] Cornett DS, Reyzer ML, Chaurand P, Caprioli RM (2007). MALDI imaging mass spectrometry: molecular snapshots of biochemical systems. Nature methods.

[16] Clemis EJ (2012). Quantitation of spatially-localized proteins in tissue samples using MALDI-MRM imaging. Analytical chemistry.

[17] Chaurand P, Norris JL, Cornett DS, Mobley JA, Caprioli RM (2006). New developments in profiling and imaging of proteins from tissue sections by MALDI mass spectrometry. Journal of proteome research.

[18] Oppenheimer SR, Wehr AY (2015). Imaging mass spectrometry in drug discovery and development. Bioanalysis.

[19] Agar NY (2010). Imaging of meningioma progression by matrix-assisted laser desorption ionization time-of-flight mass spectrometry. Analytical chemistry.

[20] Calligaris D (2013). Selected protein monitoring in histological sections by targeted MALDI-FTICR in-source decay imaging. Analytical chemistry.

[21] Fehniger TE (2011). Direct demonstration of tissue uptake of an inhaled drug: proof-of-principle study using matrix-assisted laser desorption ionization mass spectrometry imaging. Analytical chemistry.

[22] Liu H (2014). 1,5-Diaminonaphthalene hydrochloride assisted laser desorption/ionization mass spectrometry imaging of small molecules in tissues following focal cerebral ischemia. Analytical chemistry.

[23] Bottini G (1992). Oxiracetam in dementia: a double-blind, placebo-controlled study. Acta neurologica Scandinavica.

[24] Kometani M (1991). Effect of oxiracetam on cerebrovascular impairment in rats. Arzneimittel-Forschung.

[25] Baumel B (1989). Oxiracetam in the treatment of multi-infarct dementia. Progress in neuro-psychopharmacology & biological psychiatry.

[26] Gabryel B, Trzeciak HI, Pudelko A, Cieslik P (1999). Influence of piracetam and oxiracetam on the content of high-energy phosphates and morphometry of astrocytes *in vitro*. Polish journal of pharmacology.

[27] Williams K, Lee E (1985). Importance of drug enantiomers in clinical pharmacology. Drugs.

[28] Cheng AS (2013). Helicobacter pylori causes epigenetic dysregulation of FOXD3 to promote gastric carcinogenesis. Gastroenterology.

[29] Pappas BA, de la Torre JC, Davidson CM, Keyes MT, Fortin T (1996). Chronic reduction of cerebral blood flow in the adult rat: late-emerging CA1 cell loss and memory dysfunction. Brain research.

[30] Zhang Q (2015). Comparative pharmacokinetic studies of racemic oxiracetam and its pure enantiomers after oral administration in rats by a stereoselective HPLC method. Journal of pharmaceutical and biomedical analysis.

[31] Sato M, Heiss WD (1985). Effect of piracetam on cerebral blood flow and somatosensory evoked potential during normotension and hypotensive ischemia in cats. Arzneimittel-Forschung.

[32] Vlahov V, Nikolova M, Nikolov R (1980). The effect of piracetam on the local cortical cerebral blood flow in cats. Archives internationales de pharmacodynamie et de therapie.

[33] Kessler J, Thiel A, Karbe H, Heiss WD (2000). Piracetam improves activated blood flow and facilitates rehabilitation of poststroke aphasic patients. Stroke; a journal of cerebral circulation.

[34] Winblad B (2005). Piracetam: a review of pharmacological properties and clinical uses. CNS drug reviews.

[35] von Bernhardi R (2007). Glial cell dysregulation: a new perspective on Alzheimer disease. Neurotoxicity research.

[36] Abraham H, Lazar G (2000). Early microglial reaction following mild forebrain ischemia induced by common carotid artery occlusion in rats. Brain research.

[37] Buskila Y, Farkash S, Hershfinkel M, Amitai Y (2005). Rapid and reactive nitric oxide production by astrocytes in mouse neocortical slices. Glia.

[38] Liu X, Sullivan KA, Madl JE, Legare M, Tjalkens RB (2006). Manganese-induced neurotoxicity: the role of astroglial-derived nitric oxide in striatal interneuron degeneration. Toxicological sciences: an official journal of the Society of Toxicology.

[39] Badan I (2003). Accelerated glial reactivity to stroke in aged rats correlates with reduced functional recovery. Journal of cerebral blood flow and metabolism: official journal of the International Society of Cerebral Blood Flow and Metabolism.

[40] Vicente E (2009). Astroglial and cognitive effects of chronic cerebral hypoperfusion in the rat. Brain research.

[41] Matsui T (2002). Astrocytic activation and delayed infarct expansion after permanent focal ischemia in rats. Part I: enhanced astrocytic synthesis of s-100beta in the periinfarct area precedes delayed infarct expansion. Journal of cerebral blood flow and metabolism: official journal of the International Society of Cerebral Blood Flow and Metabolism.

[42] Peng Y (2007). l-3-n-Butylphthalide improves cognitive impairment induced by chronic cerebral hypoperfusion in rats. The Journal of pharmacology and experimental therapeutics.

[43] Koehler RC, Roman RJ, Harder DR (2009). Astrocytes and the regulation of cerebral blood flow. Trends in neurosciences.

[44] Ponzio F, Pozzi O, Banfi S, Dorigotti L (1989). Brain entry and direct central pharmacological effects of the nootropic drug oxiracetam. Oxiracetam: brain entry and pharmacological effects. Pharmacopsychiatry.

[45] Zhang Q (2015). Enantioselective HPLC determination of oxiracetam enantiomers and application to a pharmacokinetic study in beagle dogs. Journal of chromatography. B, Analytical technologies in the biomedical and life sciences.

[46] Durukan A, Tatlisumak T (2009). Ischemic stroke in mice and rats. Methods in molecular biology.

[47] Rastogi V (2015). Hemispheric differences in malignant middle cerebral artery stroke. Journal of the neurological sciences.

[48] Jansen van Vuuren A, Saling MM, Ameen O, Naidoo N, Solms M (2017). Hand preference is selectively related to common and internal carotid arterial asymmetry. Laterality.

[49] Gur RC (1982). Sex and handedness differences in cerebral blood flow during rest and cognitive activity. Science.

[50] Sherman GF, Garbanati JA, Rosen GD, Yutzey DA, Denenberg VH (1980). Brain and behavioral asymmetries for spatial preference in rats. Brain research.

[51] Tang AC, Verstynen T (2002). Early life environment modulates ‘handedness’ in rats. Behavioural brain research.

[52] Sa Santos S (2016). Amidated and Ibuprofen-Conjugated Kyotorphins Promote Neuronal Rescue and Memory Recovery in Cerebral Hypoperfusion Dementia Model. Frontiers in aging neuroscience.

[53] Hokonohara T, Sako K, Shinoda Y, Tomabechi M, Yonemasu Y (1992). The effects of oxiracetam (CT-848) on local cerebral glucose utilization after focal cerebral ischemia in rats. Japanese journal of pharmacology.

[54] Barsotti C, Ipata PL (2004). Metabolic regulation of ATP breakdown and of adenosine production in rat brain extracts. The international journal of biochemistry & cell biology.

[55] Cantu D, Schaack J, Patel M (2009). Oxidative inactivation of mitochondrial aconitase results in iron and H2O2-mediated neurotoxicity in rat primary mesencephalic cultures. PloS one.

[56] Tretter L, Adam-Vizi V (2000). Inhibition of Krebs cycle enzymes by hydrogen peroxide: A key role of [alpha]-ketoglutarate dehydrogenase in limiting NADH production under oxidative stress. The Journal of neuroscience: the official journal of the Society for Neuroscience.

[57] Miura D (2010). Ultrahighly sensitive *in situ* metabolomic imaging for visualizing spatiotemporal metabolic behaviors. Analytical chemistry.

[58] Choi DW, Rothman SM (1990). The role of glutamate neurotoxicity in hypoxic-ischemic neuronal death. Annual review of neuroscience.

[59] Schmidley JW (1990). Free radicals in central nervous system ischemia. Stroke; a journal of cerebral circulation.

[60] Shimada K, Jong CJ, Takahashi K, Schaffer SW (2015). Role of ROS Production and Turnover in the Antioxidant Activity of Taurine. Advances in experimental medicine and biology.

[61] Kosta P (2013). Effect of piracetam and vitamin E on phosphamidon-induced impairment of memory and oxidative stress in rats. Drug and chemical toxicology.

[62] Gupta S (2009). Reversal of propoxur-induced impairment of step-down passive avoidance, transfer latency and oxidative stress by piracetam and ascorbic acid in rats. Environmental toxicology and pharmacology.

[63] Nagafuji T, Koide T, Takato M (1992). Neurochemical correlates of selective neuronal loss following cerebral ischemia: role of decreased Na + ,K( + )-ATPase activity. Brain research.

[64] Yang GY, Chen SF, Kinouchi H, Chan PH, Weinstein PR (1992). Edema, cation content, and ATPase activity after middle cerebral artery occlusion in rats. Stroke; a journal of cerebral circulation.

[65] Sweeney MI, Yager JY, Walz W, Juurlink BH (1995). Cellular mechanisms involved in brain ischemia. Canadian journal of physiology and pharmacology.

[66] Copani A (1992). Nootropic drugs positively modulate alpha-amino-3-hydroxy-5-methyl-4-isoxazolepropionic acid-sensitive glutamate receptors in neuronal cultures. Journal of neurochemistry.

[67] O’Donnell ME, Tran L, Lam TI, Liu XB, Anderson SE (2004). Bumetanide inhibition of the blood-brain barrier Na-K-Cl cotransporter reduces edema formation in the rat middle cerebral artery occlusion model of stroke. Journal of cerebral blood flow and metabolism: official journal of the International Society of Cerebral Blood Flow and Metabolism.

[68] Taoufik E, Probert L (2008). Ischemic neuronal damage. Current pharmaceutical design.

[69] Malykh AG, Sadaie MR (2010). Piracetam and piracetam-like drugs: from basic science to novel clinical applications to CNS disorders. Drugs.

[70] Kennedy MB (1989). Regulation of synaptic transmission in the central nervous system: long-term potentiation. Cell.

[71] Ghadiri T (2014). A novel traumatic brain injury model for induction of mild brain injury in rats. Journal of neuroscience methods.

[72] Sadeghian H (2012). Neuronal death by repetitive cortical spreading depression in juvenile rat brain. Experimental neurology.

[73] Cho KO, La HO, Cho YJ, Sung KW, Kim SY (2006). Minocycline attenuates white matter damage in a rat model of chronic cerebral hypoperfusion. Journal of neuroscience research.

