# Supplementary material for: (S)-Oxiracetam is the Active Ingredient in Oxiracetam that Alleviates the Cognitive Impairment Induced by Chronic Cerebral Hypoperfusion in Rats
Source: Sci Rep. 2017 Aug 30;7:10052. doi: 10.1038/s41598-017-10283-4 (PMC5577264; doi:10.1038/s41598-017-10283-4)
Supplement: Supplementary file 1 — Supplementary data [file 41598_2017_10283_MOESM1_ESM.pdf]

Supplementary Information

(*S*)-Oxiracetam is the Active Ingredient in Oxiracetam that Alleviates the Cognitive Impairment Induced by Chronic Cerebral Hypoperfusion in Rats

Wan li [a,b], Huihui Liu[c], Hanjie Jiang [a,b], Chen Wang [a,b], Yongfei Guo [a,b], Yi Sun [a,b], Xin Zhao [a,b], Xin Xiong[d], Xianhua Zhang[d], Ke Zhang [a,b], Zongxiu Nie[c*], Xiaoping Pu [a,b*]

[a] National Key Research Laboratory of Natural and Biomimetic Drugs, Peking University, Beijing 100191, PR China

[b] Department of Molecular and Cellular Pharmacology, School of Pharmaceutical Sciences, Peking University, Beijing 100191, PR China

[c] Key Laboratory of Analytical Chemistry for Living Biosystems, Institute of Chemistry Chinese Academy of Sciences, Beijing 100190, PR China

[d] Department of Pharmacy, Peking University Third Hospital, Beijing 100191, PR China

* Corresponding authors:

Zongxiu Nie

Key Laboratory of Analytical Chemistry for Living Biosystems, Institute of Chemistry Chinese Academy of Sciences, Zhongguancun North First Street 2, Beijing, PR China, 100190. Tel: +86-10-6261-8240; Fax: +86-10-6261-8240. E-mail: znie@iccas.ac.cn

Xiaoping Pu

Department of Molecular and Cellular Pharmacology, School of Pharmaceutical Sciences, Peking University, 38 Xueyuan Rd. Haidian District, Beijing, China, 100191. Tel: +86-10-8280-2431; Fax: +86-10-8280-2431; E-mail: 11956170@pku.edu.cn

Experimental conditions of Morris water maze

The Morris water maze (SLY-ETS, Beijing Shuolinyuan Technology Co., Ltd.) was a black and circular pool (180 cm in diameter, 60 cm in height, filled to a depth of 45 cm with water at 23 ℃) that was geographically divided into four equal quadrants (Northeast, Southeast, Southwest, and Northwest) and each quadrant was marked by a different visual cue. Four points that were designed as North, South, East and West served as the starting points where the rats were gently lowered into the water, with its head facing the wall of the water maze. A circular black escape platform 10 cm in diameter (2 cm below the water surface) was placed in the center of the southwest quadrant and located at this location for escape throughout the testing period.

Preparation of matrix

To obtain 1, 5-DAN hydrochloride, 39.5 mg 1, 5-DAN was dissolved in 500 μL 1 mol/L hydrochloride and 4 mL distilled water by sonification. And then, 4.5 mL ethanol was added to the matrix solution.

Preparation of mixture standard solution

A mixture standard solution containing taurine, L-aspartate, L-glutamate, glutamine, N-acetylaspartate, ascorbic acid, citric acid, glutathione, AMP, ADP, ATP, GMP, glucose at 50 μM each was prepared in water.

Tissue Homogenate Preparation.

Brain tissue samples were weighed and homogenized (1 g: 5 m L) in 80% MeOH on ice using a ultrasonic processor. After centrifugation at 10,000 × g for 15 min at -4 ◦C, the supernatant was collected, and the precipitant was then extracted (1 g: 5 mL) with

CHCl3/MeOH (1:1, v/v) on ice by sonification for three time with an interval of 10min. Each sample was then, centrifuged at 10,000 × g for 15 min at -4◦C, the supernatant was collected. Furthermore, two parts of supernatant were mixed and dried under nitrogen. The resultant samples were stored at -80˚C. Samples were dissolved (1 g: 5 m L) in 80% MeOH and centrifuged at 18,000 × g for 10 min at -4 ◦C prior to analysis. 50 μM 4-hydroxybenzophenone were used as the internal standard in this experiment.

Supplementary Table. 1

Multiple reaction monitoring (MRM) transition and optimal DP and CE for each metabolite.

Analyte	Patent ion	Product ion	DP(V)	CE (V)
Taurine	124.1	79.8	60	30
Aspartate	131.9	87.9	50	20
Glutamine	144.9	128.1	55	15
Glutamate	145.9	102.1	40	20
N-acetylaspatate	173.9	130.1	45	18
Ascorbic acid	174.9	114.9	45	18
Glucose	179.1	89.05	80	15
Citric acid	191	110.9	50	15
Glutathione	306	143.1	80	23
GMP	362.0	211.0	70	27
AMP	346.1	133.9	100	50
ADP	426.2	134.1	90	35
ATP	506.1	158.9	100	40
4-Hydroxybenzophenone	124.1	79.8	70	40

DP (V): declustering potential, CE (V): collision energy, AMP: adenosine monophosphate, ADP: adenosine diphosphate, ATP: adenosine triphosphate, GMP: guanosine monophosphate.

Supplementary Table. 2

Peak area recorded for altered metabolites extracted from cortex of the 2-VO rats.

	Peak area			
Name	Sham	Model	Rso	So
1. Glucose (E+06)	7.92 ± 3.27	7.2 ± 2.19	10.24 ± 4.60	8.87 ± 3.57
2. Citric acid (E+06)	6.47 ± 1.40	14.94 ± 8.20*	8.83 ± 2.55	7.95 ± 2.54
3. ATP (E+03)	1.65 ± 0.44	0.81 ± 0.30**	0.76 ± 0.32	0.79 ± 0.33
4. ADP (E+05)	5.04 ± 0.68	0.83 ± 0.82***	2.94 ± 0.77**	2.57 ± 0.89**
5. AMP (E+05)	1.93 ± 0.53	0.15 ± 0.14***	0.43 ± 0.28*	0.66 ± 0.36**
6. GMP (E+05)	12.89 ± 1.65	2.43 ± 2.96***	5.96 ± 2.39*	7.12 ± 2.29*
7. Glutamate (E+07)	11.03 ± 0.95	5.47 ± 2.39***	8.14 ± 1.29*	8.43 ± 1.34*
8. Glutamine (E+06)	9.39 ± 1.99	3.38 ± 2.29***	7.23 ± 1.50**	6.87 ± 1.93*
9. Aspartate (E+07)	4.12 ± 0.54	1.35 ± 1.17***	2.31 ± 0.92	2.16 ± 0.62
10. N-acetylaspatate (E+06)	6.75 ± 0.35	1.15 ± 1.34***	3.44 ± 0.96**	3.30 ± 1.11*
11. Glutathione (E+06)	7.53 ± 2.07	0.80 ± 1.19***	3.88 ± 1.84**	3.86 ± 2.02**
12. Ascorbic acid (E+07)	9.19 ± 0.63	3.31 ± 2.20***	8.10 ± 1.08***	7.47 ± 1.77**
13. Taurine (E+07)	6.74 ± 0.56	3.64 ± 2.00**	6.52 ± 0.63*	5.81 ± 1.34
14. 4-Hydroxyben-zophenone (E+04)	3.17 ± 0.18	3.37 ± 0.06*	3.01 ± 0.23**	3.08 ± 0.24*

So: (*S*)-oxiracetam; Rso: oxiracetam. Data were presented as mean ± SD, n = 5 - 6 per group. One-way ANOVA was used to analyze the differences of groups. $^{\#\#}P < 0.01$, $^{\#\#\#}P < 0.001$ vs. sham group; $^{*}P < 0.05$, $^{**}P < 0.01$ vs. model group.

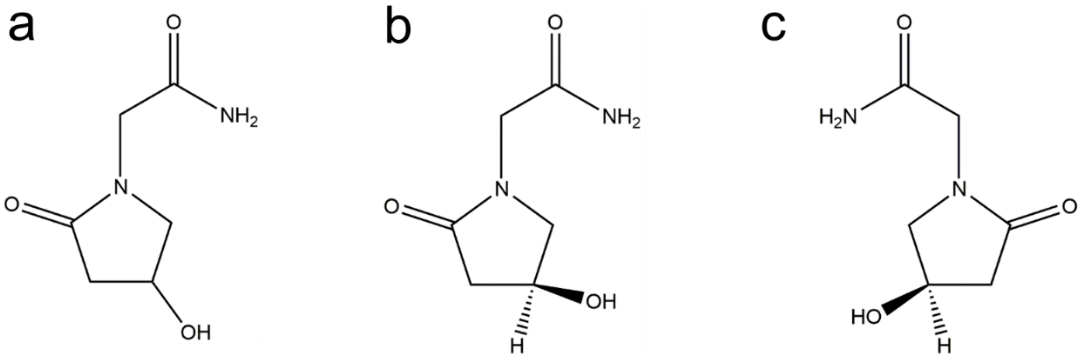

Supplementary Figure 1 | Structural formula of oxiracetam (a), (*S*)-oxiracetam (b) and (*R*)-oxiracetam (c).

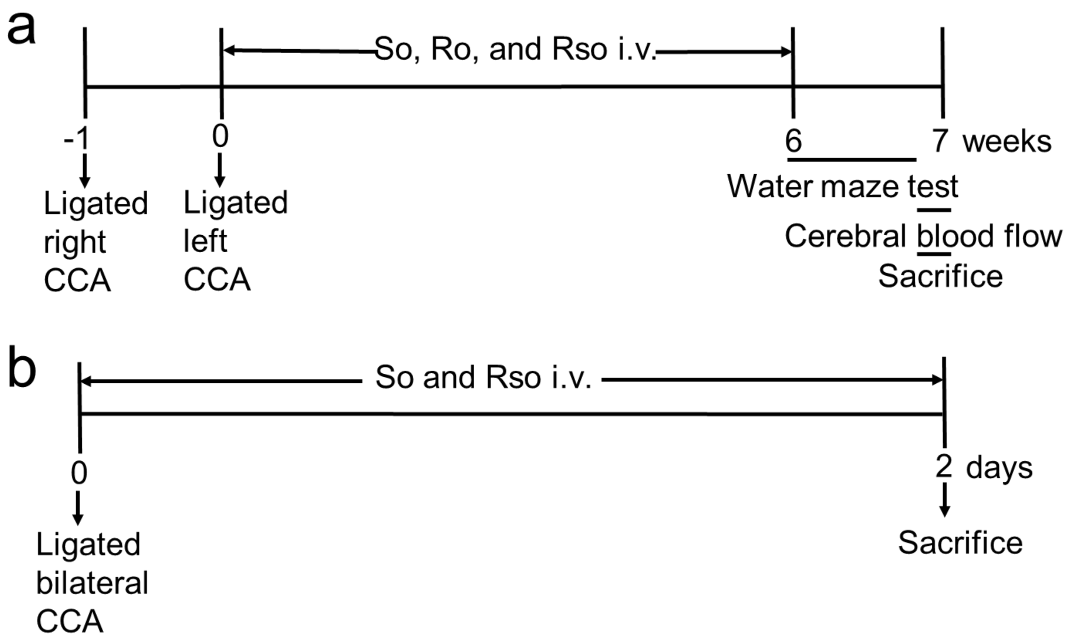

Supplementary Figure 2 | Diagrammatic representation of two experimental protocols. (a) Evaluation of the nootropic effects of S-oxiracetam in the chronic phase of 2-VO model. (b) Mass spectrometry imaging of small molecules in the acute phase of 2-VO model. So: (*S*)-oxiracetam; Ro: (*R*)-oxiracetam; Rso: oxiracetam.

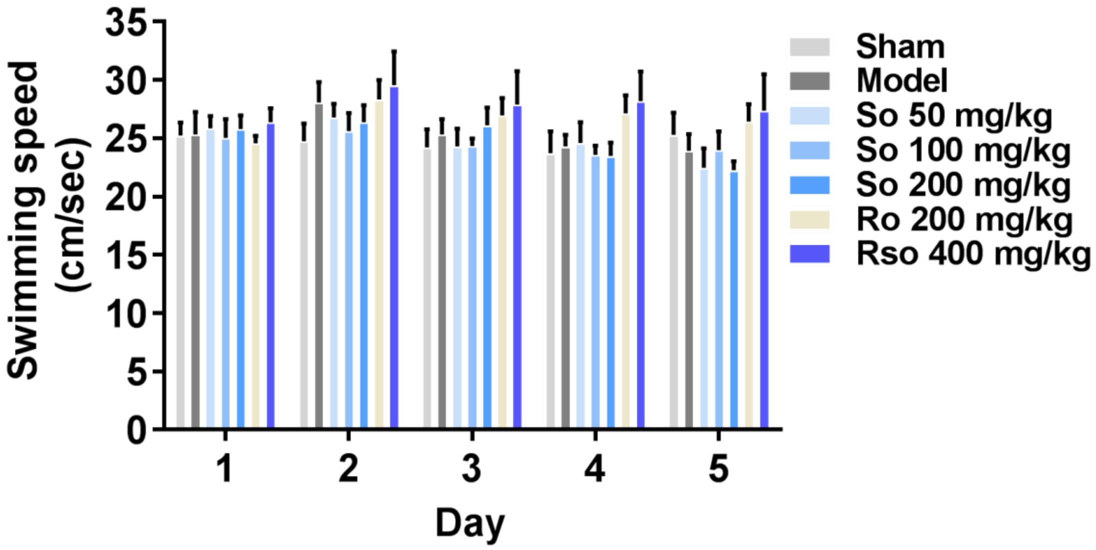

Supplementary Figure 3 | The swimming speed in the navigation test of Morris water maze test. So: (*S*)-oxiracetam; Ro: (*R*)-oxiracetam; Rso: oxiracetam. Data were presented as mean ± SEM, n = 7-9 per group. The swimming speed in the navigation training was analyzed using Two-way ANOVA of repeated measures.

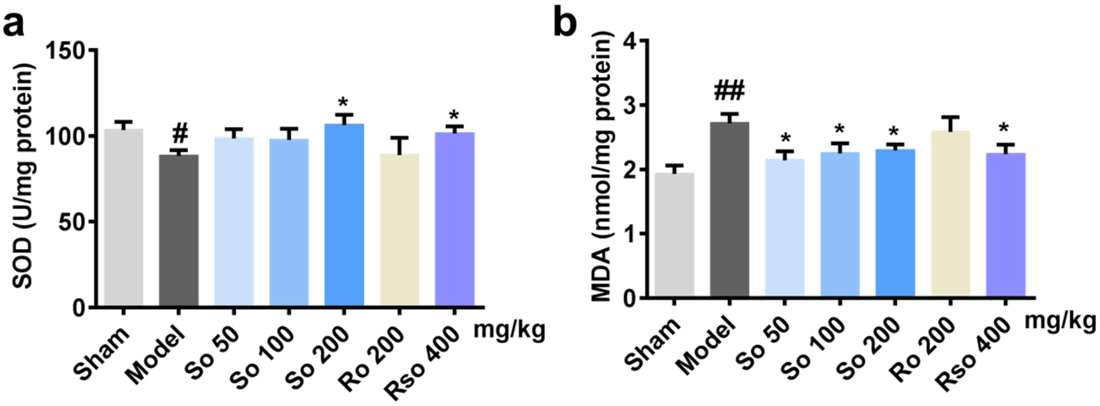

Supplementary Figure 4 | (S)- oxiracetam increased SOD activity (a) and decreased MDA content (b) in rats' cortex induced by 2-VO. So: (S)-oxiracetam; Ro: (R)-oxiracetam; Rso: oxiracetam. Data were presented as means ± SEM, n = 6-7 per group. One-way ANOVA was used to analyze the differences of groups. $^{\#}p < 0.05$, $^{\#\#}$ $p < 0.01$ vs. Sham group, * $p < 0.05$ vs. Model group.
